# Accelerated myocardial perfusion imaging using saturation-recovery spiral-in/out SSFP

**DOI:** 10.1186/1532-429X-17-S1-P408

**Published:** 2015-02-03

**Authors:** Xue Feng, Yang Yang, Craig H Meyer, Michael Salerno

**Affiliations:** 1 Biomedical Engineering, University of Virginia, Zion Crossroads, VA, USA; 2 Radiology, University of Virginia, Charlottesville, VA, USA; 3 Medicine, University of Virginia, Charlottesville, VA, USA

## Background

Myocardial first-pass perfusion imaging is a promising diagnostic tool for the assessment of ischemic heart disease. Spiral imaging, due to its high acquisition speed and robustness against motion artifacts, has been successfully combined with the saturation recovery (SR) GRE sequence using a traditional spiral-out trajectory. However, a SR SSFP sequence can provide higher SNR and use shorter saturation recovery time. In addition, as compared to a spiral-out trajectory, the spiral-in/out trajectory in an SSFP sequence can take advantage of the refocusing mechanism at TE=TR/2 to increase SNR and reduce off-resonance artifacts. Furthermore, spatial and temporal parallel imaging methods can be used to achieve up to 4x acceleration without degrading the image quality. In this study, we have implemented a spiral-in/out SR SSFP sequence and imaged rest myocardial perfusion.

## Methods

In the spiral-in/out SR SSFP sequence, a 90° SR pulse is followed by a fat sat pulse and a 40 ms SR time. The fully sampled spiral-in/out trajectory was designed with 48 interleaves and 1.6 ms readouts with a linearly decreasing sampling density to increase acquisition efficiency. Since 4x undersampling is used, 12 equally spaced interleaves were acquired at each frame and rotated by 90° for subsequent frames. To reduce the phase error, a series of 8 linearly ramping RF pulses with data acquisition starting after the first 4 pulses were applied. Two additional spiral interleaves were acquired for off-resonance field map correction. The total imaging time per slice was 110 ms.

Three patient studies were performed on a Siemens Avanto 1.5T scanner with informed consent under the local IRB. Perfusion images with 340-400 mm FOV and 1.8-2.1 mm spatial resolution at 4-6 short axis slice locations were acquired after Gd injection during an expiratory breath-hold. Chest and spine coils with up to 18 channels were used to facilitate the spatial parallel imaging technique and improve SNR. The missing k-space data was interpolated using SLAM and fed into the SPIRiT reconstruction method as an additional data constraint. The data fidelity parameters were chosen to be smaller for the interpolated k-space data than those of the acquired k-space data since the former is not as reliable as the latter.

## Results

**Figure 1:**
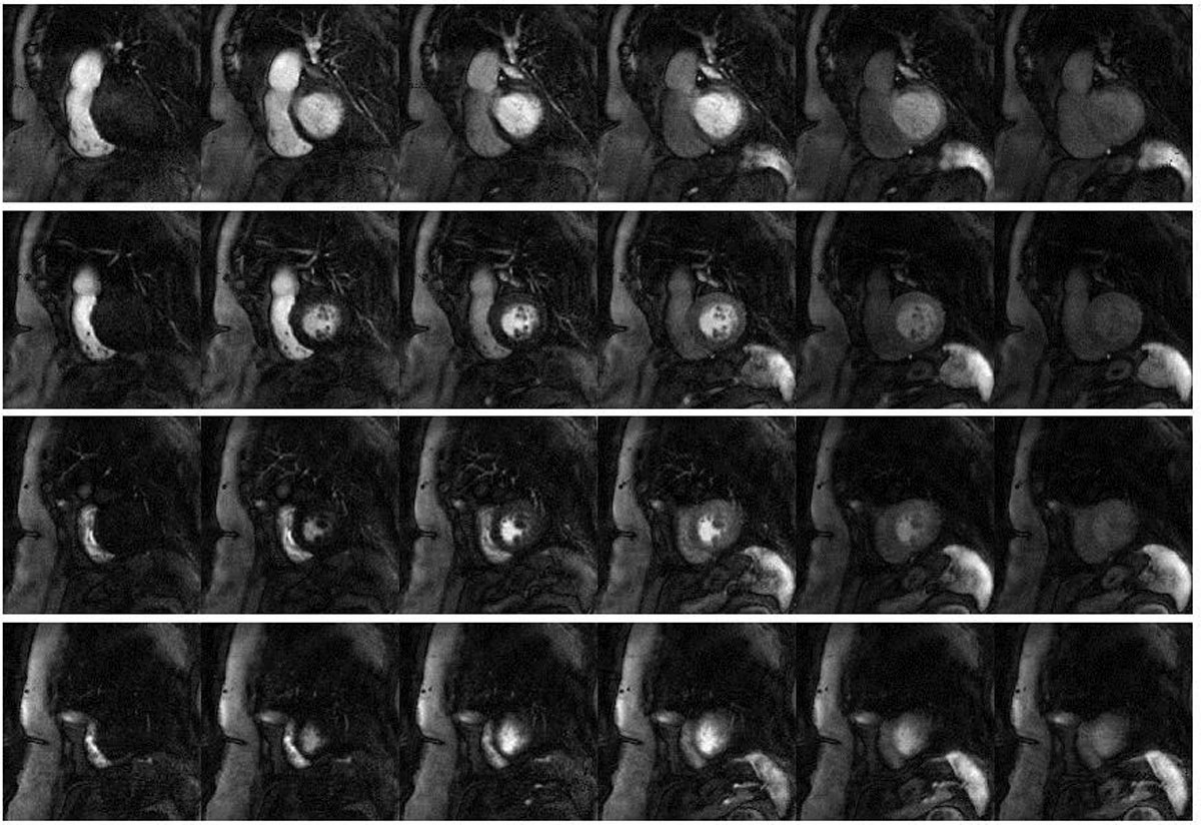
Myocardial perfusion images at every fourth frame acquired at 4 slice locations (row 1, 2, 3, 4) using the accelerated SR spiral-in/out SSFP sequence.

Fig. 1 shows a series of myocardial perfusion images acquired using the SR SSFP sequence at 4 short axis positions. Banding artifacts are minimized with the ramping RF pulses and short TR. The proposed image reconstruction method successfully eliminates the spatial aliasing with the undersampled trajectory. With the high spatial resolution (1.8mm), the coronary arteries are visible on multiple slices.

## Conclusions

We have implemented an accelerated SR SSFP sequence for myocardial first-pass perfusion imaging using the spiral-in/out trajectory. Due to its high SNR and resolution, it is a promising alternative to the traditional GRE sequence.

## Funding

NIH R01HL079110, NIH K23HL112910-02 and Siemens Medical Solutions.

